# The impact of SARS-CoV-2 measures on patient samples and complication rates in spine surgery – A single center analysis

**DOI:** 10.3389/fsurg.2022.1086960

**Published:** 2023-01-17

**Authors:** Dragan Jankovic, Harald Krenzlin, Naureen Keric, Malte Ottenhausen

**Affiliations:** Department of Neurosurgery, University Medical Center, Mainz, Germany

**Keywords:** complications, infections, SARS-CoV-2, spine, surgery

## Abstract

**Objective:**

Over the past two years during the pandemic, the German health system has taken drastic measures, like the continuous use of face masks for all staff, restrictions of hospital visits as well as cancellation of elective surgical procedures. As a potential side effect of these measures, a significant reduction of surgical site infections was reported for neurosurgical patients. The purpose of our study was to analyze the impact of these measures on spinal surgery.

**Methods:**

We performed a retrospective analysis to compare patient samples, procedures and infection rates before (January 2019 – March 2020) and during (April 2020 – June 2021) the pandemic to evaluate the impact of the measures mentioned above. Demographic and clinical data were collected and correlated with the occurrence of postoperative complications, especially infection.

**Results:**

Our analysis showed no relevant decrease of spine surgeries (838 surgeries in non-pandemic group vs. 831 surgeries pandemic group). The most common postoperative complication was wound infection in both groups, followed by urinary tract infection and pneumonia. In both patient groups, infections were more prevalent in surgeries of multilevel posterior instrumentation. Comparing the two groups of patients, a slight, non-significant (0.5%) reduction of overall postoperative complications in the pandemic group was observed. However, the number of spinal surgeries classified as emergencies in our institution increased by 10.2% during the last 15 months of the COVID-19 pandemic. In line with this finding the urgent transfer of patients from smaller hospitals increased by 14.2%, compared to previous years.

**Conclusion:**

The volume of spinal surgeries remained high and complication rates stable during the pandemic. A reason why complication rates did not drop as reported previously might be a significant change in patient sample due to the increase of emergency surgeries. A decrease of complication rates, especially infections by the measures of infection prevention for the pandemic was not observed.

## Introduction

On 30 January 2020, the World Health Organization (WHO) stated that the SARS-CoV-2 outbreak constitutes a public health emergency of international concern. On March 11, 2020, it was declared a global pandemic (1, 2). To date, 610.393.563 people have been infected worldwide, 149.169 patients died from the disease and 32.797.308 cases were diagnosed in Germany (3).

In addition to guidelines from the WHO and the Robert Koch Institute (RKI), the German Neurosurgical Society (DGNC), together with the Professional Association of German Neurosurgeons (BDNC), has issued guidelines to slow the spread of the Coronavirus SARS-CoV-2 (4). Elective surgical cases were postponed to increase the number of available hospital beds and respirators in intensive care units (5). To prevent the transmission of the virus, precautionary measures including mandatory wearing of personal protective equipment (PPE) such as surgical or FFP2 masks for medical staff and patients, wearing of disposable gloves, as well as restrictions of inpatient visits, virtual meetings and intensified cleaning protocols have been established across Germany including our institution.

Several authors reported effects of the pandemic and its countermeasures on routine neurosurgical practice. While Chacon-Quesada et al. reported a significant reduction of surgical site infections in their neurosurgical department due to the implemented measures; Abduljawwaad et al. did not note any relevant changes in patient characteristics, duration of hospital stay and mortality rates in a nationwide network of spine care centers (6, 7). An increased rate of complications in patients undergoing neurosurgery with concomitant SARS-CoV-2 infection has been by described by a group in Switzerland (8).

The aim of this study is to evaluate the impact of the SARS-CoV2 pandemic as well as common counter measures on spine surgery in a neurosurgical department at a university hospital center in the first 15 months of the pandemic.

## Material and methods

We performed a single center retrospective analysis. The institutional review board approved this study, patient consent was not required due to the retrospective nature of this study. All patients were divided into two groups. The first group of patients (non-pandemic group) represented all patients who underwent spine surgery at the Department of Neurosurgery of the University Medical Center Mainz in a 15 months period before the pandemic (from January 1, 2019 to March 31, 2020). The second group (pandemic group) was a group of patients who underwent spinal surgery in a 15 months period during the COVID-19 pandemic (from April 1, 2020 to June, 30, 2021). Preoperative and postoperative medical records as well as operative reports were reviewed. All spinal operations were included in the analysis. Emergency was defined as “requiring timely treatment within 24 h”. Triage and emergency assessment was performed by the on call neurosurgical attending. The duration of the operation was defined as the time from incision until skin closure and is reported in minutes. Surgical site location is reported as cervical, thoracic, and lumbar. Postoperative complications were divided into 6 groups: urinary tract infection (UTI), new SARS-CoV2 Infection, pneumonia, deep venous thrombosis (DVT), pulmonary embolism (PE), and wound infection which was further subdivided into superficial and deep infection. Length of stay (LOS) is reported in days.

As of hospital standard of care during the pandemic, elective patients had to be tested negative for SarsCoV2 by Polymerase Chain Reaction (PCR) test before surgery, while in case of emergency, a PCR test and a rapid antigen test were performed immediately after admission. In concordance with the WHO guidelines, all patients received preoperative prophylactic antibiotics (Cefazolin) (9). Patients with infections such as spondylodiscitis, empyema, or abscess received an antibiotic immediately after intraoperative sampling. Minor spinal procedures, such as percutaneous infiltration, lumbar punction and placement of continuous lumbar drainage were not included in this analysis.

### Statistical analysis

The frequency in differences of the investigated variables between the two groups of patients was tested by the Chi-square test, or, if necessary, by Fishers exact test. All *p* values are two-sided and the significance level is set to *α* = 0.05. MedCalc Statistical Software, version 15.11.4 (MedCalc Software bvba, Ostend, Belgium) was used for statistical analysis.

## Results

### Caseload

**Figure 1:**
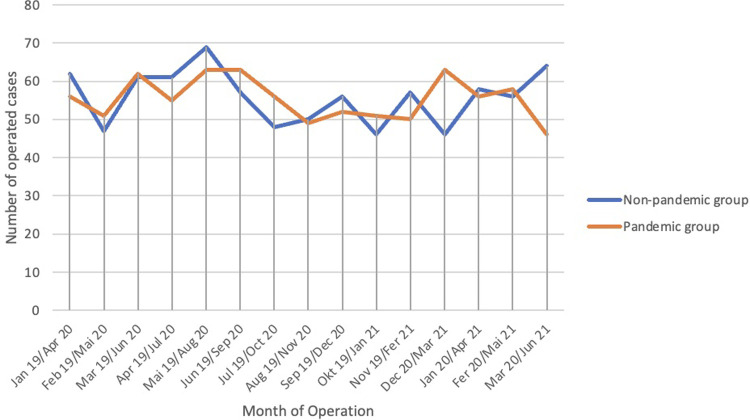
Timeline with the number of cases in the non-pandemic and pandemic group.

During the 15 months between January 1, 2019 and March 31, 2020, a total of 838 spine surgeries were conducted. In the period between April 1st 2020 and June 31st 2021 the department performed 831 spinal procedures (Figure 1).

During the first period 19% of all procedures were classified as emergencies while during the second period 29.2% were classified as emergency procedures.

### Patient sample

**Figure 2:**
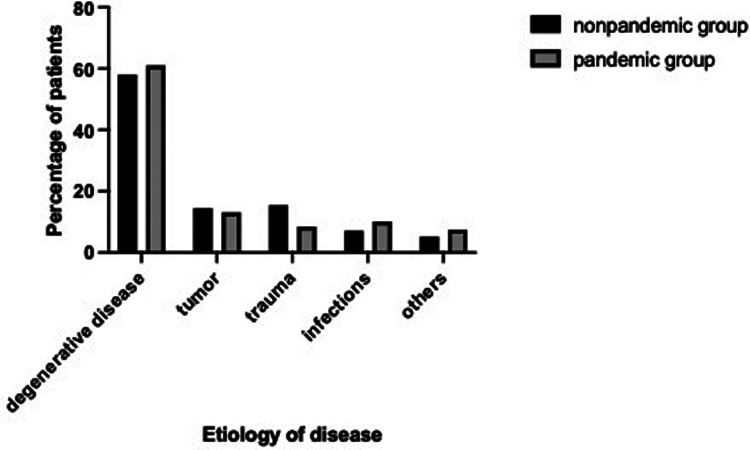
Distribution of disease etiology in non-pandemic and pandemic group.

During the first period 838 operations were performed on 810 patients of those 346 (42.7%) were female, 464 (57.3%) were male. The mean age of patients was 61.8 years (2–95 years). Reasons for operation were degenerative disease in 470 patients (58%), tumor in 117 patients (14.4%), trauma in 124 patients (15.4%), infection in 57 patients (7.1%) and others in 42 patients (5.2%) (Figure 2). A total of 436 (52%) operations were performed involving the lumbar spine, 167 (20%) the thoracic spine and 235 (28%) the cervical spine. 344 (41%) patients received dorsal instrumentation, 689 (82.2%) *via* a posterior approach, 109 (13%) *via* an anterior and 40 (4,8%) through a lateral approach.

In the second period, a total of 831 procedures were performed on 808 patients. 384 (47.5%) were female, 424 (52.5%) were male. The mean age of patients was 59.1 years (range 2–93 years). Indications for operation included degenerative disease in 493 patients (61%), tumor in 106 patients (13.1%), trauma in 67 patients (8.4%), infection in 82 patients (10.1%) and others in 60 patients (7.4%) (Figure 2). A total of 407 (49%) operations were performed involving the lumbar spine, 200 (24%) the thoracic spine and 224 (27%) the cervical spine. 382 (46%) received instrumentation, 623 (75%) *via* a posterior approach, 133 (16%) *via* an anterior and 75 (9%) through a lateral approach Table 1. shows a comparison of the patient samples of the two periods.

**Table 1:** Comparison of the patient samples of the two periods.

	Non-pandemic group	Pandemic group
**Male**	57.3%	51%
**Female**	42.7%	49%
**Mean Age (years)**	56.8	54.4
**Indication for surgery**		
Degenerative disease	58%	61%
Tumor	14.4%	13.1%
Trauma	15.4%	8.4%
Infections	7.1%	10.1%
Others	5.2%	7.4%
**Location**		
Cervical	28%	27%
Thoracic	20%	24%
Lumbar	52%	49%
**Approach**		
Posterior	82.2%	75%
Anterior	13%	16%
Lateral	4.8%	9%
***Instrumentation***	41%	46%

### Complication rate

**Figure 3:**
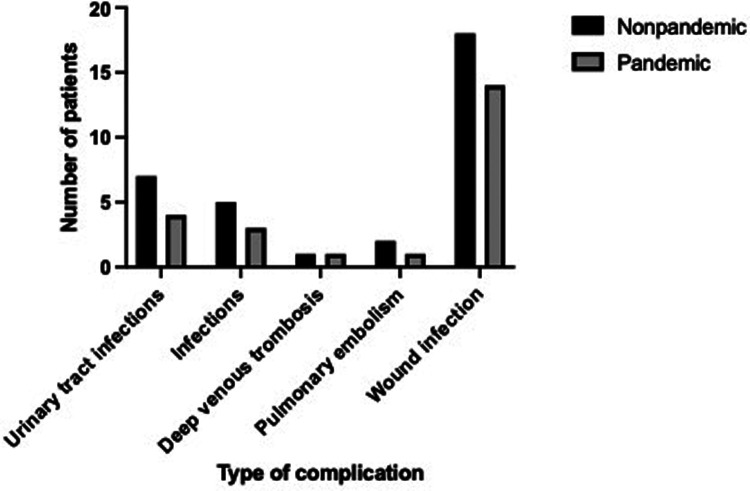
Distribution of postoperative complications.

The overall complication rate in the period before the pandemic was 3.9% (*n* = 33). Of these 33 patients, 9 (27.3%) underwent emergency surgery, and 24 (72.7%), elective surgery (Table 2). The most common postoperative complication in the non-pandemic group was wound infection with a prevalence of 2.4%, followed by urinary tract infections with a prevalence of 0.95% and pneumonia, which occurred in 0.67% of patients. Pulmonary embolism occurred in 2 patients, while 1 patient had a deep vein thrombosis (Figure 3).

**Table 2:** Patients with complications.

	Nonpandemic group (*n* = 33)	Pandemic group (*n* = 24)
**Age**	68.70 ± 12.00	62.75 ± 13.27
**Gender (male:female)**	1,36:1	1:2
**ASA Class**		
1	8 (24.2%)	3 (12.5%)
2	11 (33.3%)	10 (41.7%)
3	13 (38.2%)	10 (41.7%)
4	1 (3%)	1 (4.2%)
**Type of operation**		
Emergency	9 (27.3%)	9 (37.5%)
Elective	24 (72.7%)	15 (62.5%)
**Approach**		
Anterior	3 (9.1%)	0%
Posterior	30 (90.9%)	24 (100%)
**Location**		
Cervical	2 (6.1%)	0%
Thoracal	10 (30.3%)	6 (25%)
Lumbal	21 (63.6%)	18 (75%)
**Levels of operation**		
1 level	9 (27.3%)	6 (25%)
2 levels	11 (33.3%)	7 (29.2%)
3 An more levels	13 (39.4%)	11 (45.8%)
**Comorbidites**		
Obesity	11 (33.3%)	8 (33.3%)
Diabetes Mellitus	5 (15.2%)	4 (16.7%)
Smoking	8 (25.0)	4 (16.7)
**Duration of surgery**	172.30 ± 50.80	159.3 ± 52.10
**Lengh of stay**	15.27 ± 10.51	15.8 ± 12.3

Preoperatively, respectively 38.2% of patients were classified as ASA III (*n* = 13) and 33.3% as ASA II (*n* = 11), followed by ASA I (24.2%) and only one patient classified as ASA IV.

During the 15 months period after the introduction of intensified cleaning protocols in April 2021 and restrictions of hospital visits, 831 spinal surgeries were performed. The prevalence of deep wound infection in the pandemic group was 1.9%, the prevalence of urinary tract infection and pneumonia was 0.54% each. The distribution of species of microorganism causing postoperative infections is shown in Table 3.

**Table 3:** Distribution of species of microorganism causing postoperative infections.

	Prepandemic cohort			Pandemic cohort		
	*UTI*	*Pneumonie*	*Wound infection*	*UTI*	*Pneumonie*	*Wound infection*
Gram-negative	4	2	5	3	2	4
Gram- positive	1	0	8	0	0	6
Mixed fluor	2	0	2	1	0	0
No detected	0	3	3	0	2	4

Only one patient with a positive SARS CoV2 infection was operated on urgently. There were no significant complications in the postoperative course. In four patients with a positive SARS CoV2 infection, elective surgery was canceled until the SARS CoV2 infection was resolved. In the second time interval, 41.7% (*n* = 10) of patients were classified as ASA II and ASA III, followed by ASA I (*n* = 3/12.5%) and ASA IV (*n* = 1/4.2%).

Patients who had a complication in the pandemic group were slightly younger than in the nonpandemic group (62.7 vs. 68.7, *P* < 0.001).

In both patient groups, infections were more prevalent in surgeries *via* a posterior approach (90.9% in the non-pandemic group vs. 100% in the pandemic group; *P* = 0,607). Postoperatively, single level operations had a lower complication rate than multilevel operations.

## Discussion

Our analysis showed that although the number of operations in the pandemic group remained stable, there is a dynamic in the number of emergency operations. To our best knowledge, this is one of the first studies that compares the pre-pandemic and pandemic period and the dynamic of operative procedures as well as perioperative complications.

Given the strain on the health care system during the pandemic and the need for additional intensive beds and staff, the number of surgical procedures had been reduced worldwide (10, 11). During the peak of the pandemic, more than 100,000 surgeries were canceled per week in the United States (12). A survey of 25 neurosurgical departments in Europe conducted by Mathiesen et al. reported that neurosurgical activity was reduced in 80% of responding centers and Tamburelli et al. reported a 50% reduction of surgical procedures during the COVID-19 pandemic (13, 14). Sivakanthan and colleagues reported a significant impact of COVID-19 on surgical productivity with total work relative value units production decreased as much as 75% (15).

A study conducted by Mehta and Chiu showed a decrease in spine cases by 63% in 2020 compared to 2019 (16). In contrast to these reports, our analysis showed no decrease in spine surgeries during the pandemic in our department.

As surgical departments in smaller hospitals were partially or completely closed, an increase in emergency transfers from smaller hospitals was observed. Compared to previous years, there was an increase of 14.2% in emergency transfers. It is such, that a trend towards higher numbers of emergency procedures during the SARS-CoV2 pandemic was observed. Comparing the two periods, a 10.2% increase in emergency operations was documented in the pandemic group which can be explained by the above-mentioned increased admission of emergency patients from smaller hospitals and is in line with findings by de Bock et al. who reported an increase in the number of emergency surgeries during the COVID-19 outbreak and with Abduljawwad et al. who reported no significant change in the prevalence of fusion procedures during the pandemic (7, 17).

A recent study from our institution reported a decrease in emergency admissions of spinal emergencies by 30.9 ± 14.6% during the first month of COVID-19 lockdown (18) An observational retrospective study conducted by Baugh et al. reported a 30.9% reduction in emergency room visits between March 2019 and March 2020 (19). Reasons for receding number of emergency visits during the pandemic could be delayed diagnosis, which might ultimately result in increased complication rates and worse outcomes. So far, we have not observed that.

In our study, ASA 2 and 3 were equally prevalent in both cohorts of patients. Sebaaly et al. reported that higher ASA score is associated with increased risk of SSI (20). Comorbidities such as obesity, smoking, and diabetes mellitus were also equally distributed between the studied cohorts of patients, with obesity being the dominant comorbidity for the development of complications in both groups. The most common postoperative infections include wound infection, pneumonia, urinary tract infection, and sepsis. These types of infection are known to be associated with an increased risk of postoperative morbidity and prolonged hospitalization (21).

### Complication rate

A study conducted by the COVIDSurg Collaborative reported that routine preoperative testing for SARS CoV2 is associated with lower postoperative pulmonary complications in major surgeries (22).. In addition, Greuter et al. reported a higher rate of postoperative thrombotic complications in patients undergoing neurosurgical procedures (8). This is explained by the state of hypercoagulation with increased rate of heparin-binding proteins, which led to an increased rate of thrombotic complications.

Comparing our two cohorts, a 0.5% reduction in postoperative wound infections was observed within the pandemic cohort without reaching statistically significance. The other types of complications (UTI, pneumonia) were also slightly decreased (0.49% vs. 0.13%, *P* = 0,150). Considering that the intensification of hygienic measures, restriction of visits and additionally strengthened postoperative care were the only novelties that were introduced in the past year, this might have contributed to a slight decline in postoperative infections.

At the beginning of the pandemic, the postponement of elective surgeries was suggested due to complications related to COVID 19 (23). Given that certain cancer patients during the non-pandemic period have shown that delayed surgical treatment is associated with worse overall survival, a balance is needed between the risk of postoperative complications and the risk of overall worse outcomes due to the primary disease (24). The patients who had concomitant comorbidities had postoperative complications or were more susceptible to postoperative infections (25). A large population study conducted in the United States that included 5,479 patients showed that patients who underwent surgery near the time of infection had an associated risk of postoperative pneumonia, respiratory failure, pulmonary embolism, and sepsis. A surgical procedure performed 8 weeks after surgery is not associated with an increased risk of postoperative complications (26). Furthermore, Baiochi et al. reported that asymptomatic patients with COVID 19 had no associated risk of postoperative complications compared to patients with a negative preoperative test for COVID 19 (27). We believe that a comprehensive preoperative assessment of SARS CoV2 status is extremely important to ensure optimal clinical decision-making.

There are several limitations of this study. Our study is a retrospective study of a single center. The study periods are designed according to the establishment of enhanced measures in our institution due to the pandemic. Therefore, in this study there is a pre-pandemic group - 15 months before the pandemic, that is, from January 2019 to March 2020, and a pandemic group in which patients underwent surgery from April 2020 to June 2021. Although the term “emergency surgery” is defined as surgical treatment within 24 h, a few emergency patients underwent surgery after 24 h because of the increased risk of bleeding due to double or triple anticoagulant therapy. However, we believe that our study can represent a solid basis for further research.

## Conclusion

During the pandemic the total number of spine surgeries in our institution did not decrease. Our analysis showed an increased number of emergency surgeries, as well as unaltered complication rates. Despite the challenges, the quantity and quality of spinal procedures could be maintained throughout the pandemic.

## Data Availability

The original contributions presented in the study are included in the article/Supplementary Material, further inquiries can be directed to the corresponding author/s.
